# Effectiveness of personalised, home-based nutritional counselling on infant feeding practices, morbidity and nutritional outcomes among infants in Nairobi slums: study protocol for a cluster randomised controlled trial

**DOI:** 10.1186/1745-6215-14-445

**Published:** 2013-12-27

**Authors:** Elizabeth W Kimani-Murage, Catherine Kyobutungi, Alex C Ezeh, Frederick Wekesah, Milka Wanjohi, Peterrock Muriuki, Rachel N Musoke, Shane A Norris, Paula Griffiths, Nyovani J Madise

**Affiliations:** 1 African Population and Health Research Center (APHRC), P.O. 10787, 00100 Nairobi, Kenya; 2 Department of Paediatrics, University of Nairobi, Nairobi, Kenya; 3 MRC/Wits Developmental Pathways for Health Research Unit, Faculty of Health Sciences, University of the Witwatersrand, Johannesburg, South Africa; 4 Centre for Global Health and Human Development, Loughborough University, Loughborough, UK; 5 Centre for Global Health, Population, Poverty, and Policy University of Southampton, Southampton, UK

**Keywords:** Breastfeeding, Infant feeding practices, Child nutrition, Cluster randomised controlled trials, Kenya, Sub-Saharan Africa, Urban slums

## Abstract

**Background:**

Nutrition in the first 1,000 days of life (during pregnancy and the first two years) is critical for child growth and survival. Poor maternal, infant and young child nutrition (MIYCN) practices are widely documented in Kenya, with potential detrimental effects on child growth and survival. This is particularly a problem in slums, where most urban residents live. For example, exclusive breastfeeding for the first six months is only about two per cent. Innovative strategies to reach slum residents are therefore needed. Strategies like the Baby Friendly Hospital Initiative have proven effective in some settings but their effectiveness in resource-limited settings, including slums where many women do not deliver in hospital, is questionable. We propose to test the effectiveness of a home-based intervention on infant feeding practices, nutrition and health outcomes of infants born in two slums in Nairobi, Kenya.

**Methods/Design:**

The study, employing a cluster-randomised study design, will be conducted in two slums in Nairobi: Korogocho and Viwandani where 14 community units (defined by the Government’s health care system) will form the unit of randomization. A total of 780 pregnant women and their respective child will be recruited into the study. The mother-child pair will be followed up until the child is one year old. Recruitment will last approximately one year and three months from September 2012 to December 2013. The mothers will receive regular, personalised, home-based counselling by trained Community Health Workers on MIYCN. Regular assessment of knowledge, attitudes and practices on MIYCN will be done, coupled with assessments of nutritional status of the mother-child pairs and diarrhea morbidity for the children. Statistical methods will include analysis of covariance and multinomial logistic regression. Additionally, cost-effectiveness analysis will be done. The study is funded by the Wellcome Trust and will run from March 2012 to February 2015.

**Discussion:**

Interventions aimed at promoting optimal breastfeeding and complementary feeding practices are considered to have high impact and could prevent a fifth of the under-five deaths in countries with high mortality rates. This study will inform policy and practice in Kenya and similar settings regarding delivery mechanisms for such high-impact interventions, particularly among urban poor populations.

**Trial registration:**

ISRCTN83692672

## Background

Despite reduction of undernutrition being one of the Millennium Development Goals (MDGs) targets, undernutrition continues to be a great public health concern in many developing countries, particularly in sub-Saharan Africa (SSA) [1]. Millions of children globally suffer from undernutrition despite many declarations and action plans aimed at combatting the phenomenon. A total of 165 million, 101 million and 51 million children under the age of five are estimated to be stunted, underweight and wasted, respectively. What is more worrying is the high level of malnutrition in SSA, where approximately 40% of all children aged less than five years (56 million) are estimated to be stunted [2]. Undernutrition is a serious risk factor for ill health, and contributes substantially to the burden of disease in developing countries. It is associated with more than half of all child deaths, and is responsible for 15% of the total loss in disability-adjusted life years (DALYs) in countries with high child mortality [3,4]. Increasingly, adverse ramifications of childhood undernutrition are recognized later in the life course. These include impaired cognitive development and poor educational achievement; low economic productivity; obesity in adulthood and a consequent risk for metabolic disease [5-8].

There is a growing recognition of the importance of nutrition in the first 1,000 days of life (during pregnancy and the first two years) with regard to child growth, health and survival. In 2002, the World Health Organization (WHO) and the United Nations Children’s Fund (UNICEF) jointly developed the global strategy for infant and young child nutrition (IYCN), which aims to revitalize efforts to promote, protect and support appropriate infant and young child feeding, thereby working towards alleviating the burden of disease associated with malnutrition among the world’s children [9]. The strategy builds on past initiatives such as the Innocenti Declaration of 1990 and the Baby-friendly Hospital Initiative (BFHI) launched in 1991. The WHO recommends exclusive breastfeeding in the first six months of life, followed by extended breastfeeding for two years or beyond, coupled with timely, nutritionally adequate, safe and appropriately fed complementary foods for optimal growth of the child. The global strategy on IYCN highlights the notion that inadequate knowledge about proper foods and feeding practices is often a more important determinant of malnutrition than the availability of food [9,10]. It is estimated that interventions that promote optimal breastfeeding and complementary feeding could prevent about a fifth of the under-five deaths in countries with high mortality rates [11,12].

Poor breastfeeding and complementary feeding practices have been widely documented in the low- and middle-income countries (LMICs), where only about 39% of infants are exclusively breastfed for the first six months. In Kenya, like in other LMICs, although almost all children are breastfed, poor maternal, infant and young child nutrition (MIYCN) practices are widely documented. However, some recent positive change in early infant feeding practices has occurred as in the 2008/2009 Kenya demographic and health survey (KDHS), 32% of children are recorded as exclusively breastfed for six months, an improvement from 13% in 2003, as reported in KDHS 2003 [13,14]. Additionally, only 39% of children aged 6 to 23 months are fed according to IYCN guidelines [15,16], and maternal nutrition is widely influenced by cultural beliefs and practices. Consequently, substantial levels of child malnutrition and poor child health and survival have been documented in Kenya, including high levels of stunting (35%) among children less than five years of age [13].

To address the problem of child malnutrition, the Kenyan Government developed a strategy in 2007, mirroring the WHO/UNICEF global strategy for IYCN, aimed at promoting optimal IYCN practices in the country [9,17]. The strategy is implemented mainly through the BFHI, which promotes breastfeeding in maternity wards around the time of delivery. Although there has been some improvement in the proportion of children exclusively breastfed in the last few years, the impact of this hospital-based initiative in low-income countries like Kenya is questionable. This is because most women, especially the poor, deliver at home and IYCN practices are greatly influenced by traditional beliefs and practices [13,18]. For example, in Kenya, only 42% of women deliver in health facilities, and this has not changed much in the last 20 years [13].

Urban poor settlements are expanding at an alarming rate in SSA, with the majority of urban residents living in slum settlements [19], thus presenting a unique challenge with respect to implementing the IYCN strategy in Kenya. These slums have been marginalized with regard to provision of Government services; hence public health facilities are rare. Additionally and consequently, a substantial proportion of slum women do not deliver at health facilities, while others deliver in sub-standard private health facilities; only 48% deliver in facilities with skilled birth attendants [20,21]. This means that many of these women do not benefit from the counselling on IYCN offered through BFHI. Findings from studies done in the urban slums of Nairobi indicate high levels of suboptimal MIYCN practices and malnutrition. For example, only two per cent of infants are exclusively breastfed for the first six months, while 15% of infants stop breastfeeding by the end of one year [22]. Furthermore, about four in ten children under the age of five are stunted [23]. This level of malnutrition in an urban setting may not entirely be due to poverty but may reflect practices that might be addressed with proper counselling and advice.

The aim of this cluster randomised controlled trial is to test the effectiveness of personalised, home-based nutritional counselling of pregnant and lactating mothers by Community Health Workers (CHWs) on MIYCN practices, and consequently on morbidity and nutritional outcomes of infants in two Nairobi slums. Specifically, we aim to determine: (i) the effect of the intervention on breastfeeding and complementary feeding knowledge, attitudes and practices among mothers of infants; (ii) the effect of the intervention on nutritional outcomes among infants; (iii) the effect of the intervention on morbidity from diarrhea among infants; (iv) the experiences, and facilitating and limiting factors associated with the intervention; and (v) the cost-effectiveness of the intervention. The primary objective is to change breastfeeding practices, particularly to improve the rate of exclusive breastfeeding, which is currently extremely low in the slums [22], despite its documented importance in child survival [11]. The primary hypothesis is that personalised, home-based nutritional counselling and support of mothers in Nairobi slums by CHWs will lead to higher knowledge and self-efficacy in breastfeeding, thereby resulting in adherence to WHO guidelines for breastfeeding. This is expected to lead to improved rates of exclusive breastfeeding for six months. The CHWs will therefore counsel mothers on what they should do regarding breastfeeding and other maternal MIYCN practices and how they should do it including breast positioning and attachment and expressing breast milk, which is expected to lead to higher knowledge and self-efficacy. The proposed strategy (community-based initiative), backed by the Ministry of Health, builds on the existing MIYCN strategy by the Government and is expected to get over the problem of women having to present at hospitals to receive information. This is important for urban poor women who have limited health care access [20,21]. The backing by the Government is critical for potential scale-up of the strategy within the Kenyan health care system if it proves to be successful in the trial.

## Methods/Design

### Study setting

**Figure 1:**
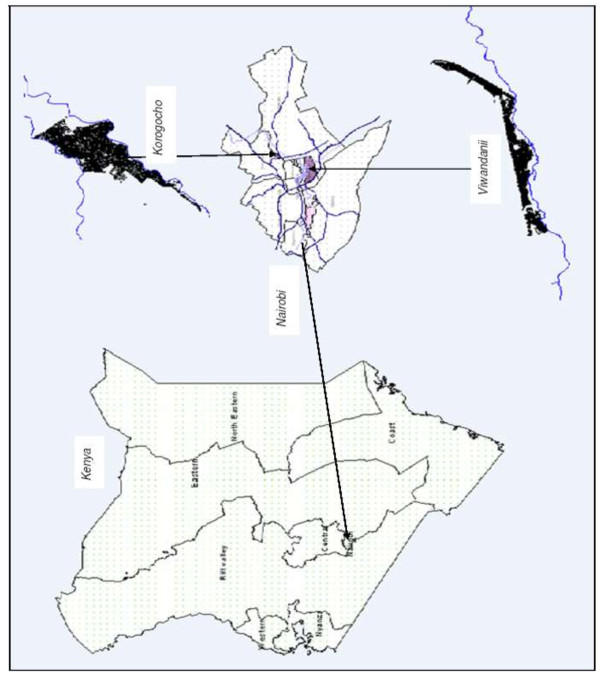
**Maps of the study areas (Korogocho and Viwandani).** Source: Emina *et al*. 2009 [25].

The study will be carried out in two slums of Nairobi, Kenya (Korogocho and Viwandani) where the African Population and Health Research Center (APHRC) runs the Nairobi Urban Health and Demographic Surveillance System (NUHDSS), covering close to 70,000 residents (Figure 1). The two slum areas are densely populated with 63,318 and 52,583 inhabitants per square km, respectively, and are characterized by poor housing, lack of basic infrastructure, violence, insecurity, high unemployment rates, and poor health indicators [24]. The two slums are located about 7 km from each other. The NUHDSS involves a systematic quarterly recording of vital demographic events, including births, deaths and migrations occurring among residents of all households in the NUHDSS area since 2003. Other data, including household assets, morbidity, and education among others, are also collected and updated regularly. More information regarding the study area and the NUHDSS can be obtained from previous publications [25].

### Study design and randomization

The study will adopt a cluster-randomised trial design [26,27]. For pragmatic purposes, community units (CUs), defined by the Government’s Community Strategy, will be used as the clusters. Fourteen CUs have been defined in the study areas: eight in Korogocho and six in Viwandani. These CUs will be randomised with half allocated to the intervention and the other half to the control arm. The random sequence of allocation of the CUs to the intervention or control arm will be computer-generated. The clusters will be stratified with regard to two aspects before randomization: slum of residence (Korogocho and Viwandani) and number of women of reproductive age in each cluster: large clusters and smaller clusters. Those in the same slum and in the same strata with regard to size will be randomly allocated to either intervention or control group. Cluster randomization is preferred over individual-level randomization to minimize contamination and for pragmatic purposes in case of future scale-up of the intervention. Randomization will be done by a data analyst who is not a primary member of the study team.

### Clusters and study populations

Community units (CUs) in Korogocho and Viwandani that fall within the NUHDSS area will constitute the clusters to be included in the study. CUs are geographically defined units, mostly equal to a village (in our study setting) and usually have a population size of approximately 5,000 people. The CUs are defined by the Community Strategy, a Government community-based approach where CHWs provide health care services to people at the community level. The reason for choosing to work with the defined CUs is pragmatic because they are administrative areas that are defined by the health care system and any intervention moving forward in the future would need to map onto these administrative units to be effective.

Study participants will include all pregnant women aged between 12 to 49 years old, who are residents of CUs in Korogocho and Viwandani slums that fall within the NUHDSS area, and their respective children (when born). These will be recruited during pregnancy on a rolling basis until the desired sample size is achieved. The target is to recruit the women as early as possible during pregnancy, particularly during the first or second trimester. While the reproductive age in most studies is usually defined as 15 to 49 years, we include girls aged 12 to 14 years because a substantial proportion (close to 10%) of adolescents in the study areas is sexually active before the age of 15 years [28].

To be excluded from the study will be: (a) women of reproductive age who will have given birth before the proposed intervention starts; (b) women with a disability that makes delivery of the intervention difficult (for example, a hearing or sight problem, or mental handicap).

To be dropped from the analysis will be: (a) women who lose the pregnancy and/or have a still-birth; (b) women who are lost to follow-up during pregnancy; and (c) mother-child pairs of children with a disability.

### Recruitment

Recruitment of participants will be done through routine NUHDSS rounds whereby pregnancy registration is done for female residents aged 12 to 49 years in each household. This will be complemented by other means of identification of pregnant women including through antenatal care providers in the study areas and use of community informants to ensure high coverage. All known pregnant women in the two study slums that fall within the Nairobi Urban Health and Demographic Surveillance Area will be invited to be recruited to the study until the desired sample size is achieved. Recruitment is expected to be for a period of approximately one year and three months from September 2012 to December 2013.

### Sample size considerations

The sample size determination was done considering the study design of cluster randomisation since villages in the study sites will be randomised to either control or intervention [29]. A minimum sample size for both intervention and control arms of 196 was estimated to have enough power to detect an increase in exclusive breastfeeding for six months from two per cent (the baseline rate of exclusive breastfeeding in the study setting) [22] to 12%; a ten percentage point increase, although higher increase has been documented in similar interventions elsewhere in the developing world [30]. We used a level of precision of five per cent (for a two-sided *t*-test) and power of 80%. We then adjusted for expected intra-cluster correlation (ICC) using a design effect of 3.2. The design effect was based on an ICC of 0.05, based on previous research on breastfeeding practices in the study area which acts as a baseline for the current study. We allowed for 20% potential attrition, also based on previous research in the study area [31]. Therefore, an effective sample size of about 780 mother-child pairs was estimated. We used practice of exclusive breastfeeding to undertake the sample size calculations as it is our primary outcome of interest. (More detailed account of sample size determination is attached as an additional material in Additional file 1).

### Intervention

The intervention will involve personalised, home-based counselling of pregnant women and mothers of infants on optimal MIYCN practices by CHWs Table 1.

**Table 1:** Intervention versus control group

**Intervention group**	**Control group**
A) Personalised, home-based counselling of mothers on maternal, infant and young child nutrition (MIYCN) (intervention)	A) **Not Provided**
B) Distribution of MIYCN educational materials (usual care)	B) Distribution of MIYCN educational materials (usual care)
C) Home-based counselling by community health workers (CHWs) on usual care (for example, ante-natal care, delivery with skilled attendants, immunization)	C) Home-based counselling by CHWs on usual care (for example, ante-natal care, delivery with skilled attendants, immunization)

A formative study will be conducted before the roll-out of the intervention to inform the design and components of the intervention including content of the counselling messages. This will involve a qualitative study involving interviews with key informants in the study communities including community leaders, CHWs, traditional birth attendants (TBAs) and health professionals; mothers (pregnant, breastfeeding and mothers of children under five years. Additionally, consultations will be held with key organizations including the Division of Nutrition and the Division of Community Health Services in the Ministry of Health; UNICEF and other organization working on MIYCN issues. The information gathered will be used to adapt the counselling messages and information materials. The formative study will also establish mechanisms for successfully engaging CHWs into the study. More details on data collection are given below.

Existing CHWs within the Government’s Community Strategy in the study area will be trained on counselling of appropriate MIYCN practices using The Community Infant and Young Child Feeding (IYCF) Counselling Package developed by UNICEF in partnership with other organizations, which has been adopted by the Kenya Ministry of Health (http://www.unicef.org/nutrition/files/Facilitator_Guide_September_2011_clean.pdf).

The elements of counselling package are based on WHO/UNICEF IYCF guidance documents, training and other materials, including the Infant and Young child feeding counselling integrated course [32]. The package is designed to equip community workers or primary health care staff to be able to support mothers, fathers and other caregivers to optimally feed their infants and young children. The CHWs will be equipped with infant and young child feeding counselling cards; brightly coloured illustrations that depict key infant and young child feeding concepts and behaviours for the CHWs to share with mothers, fathers and other caregivers. The package will be adapted to include counselling messages on maternal nutrition.

Counselling will be initiated during pregnancy as soon as the mother is recruited and will be continued until the end of infancy (one year after delivery). Counselling will encompass maternal nutrition, immediate initiation of breastfeeding after birth, breast positioning and attachment, exclusive breastfeeding, frequency and duration of breastfeeding, expressing breast milk, storage and handling of expressing and lactation management. It will also focus on age-appropriate complementary feeding, starting at six months: age appropriate complementary foods (nutritious, safe, affordable, and locally available), feeding frequency and quantity, and appropriate feeding practices including hygiene and responsive feeding behaviours, which encourage mother-child interaction during feeding [33,34]. For the intervention arm, CHWs will visit the pregnant woman about once every month up to week 34, after which they will visit the mother weekly until delivery. After delivery, they will visit the mother weekly in the first one month, then once a month until the end of infancy, but during the fifth month when mothers are expected to initiate complementary feeding, the frequency will be biweekly.

All recruited pregnant women in both the intervention arm and control arm will receive information materials regarding MIYCN throughout the follow-up period; these are normally provided by the Ministry of Health for distribution as part of the standard care. Additionally, the recruits will be provided with standard care counselling by CHWs, which will address antenatal and postnatal care, appropriate tests during pregnancy, health facility delivery, general nutrition, hygiene, and immunization. Women in the control arm will be visited according to the usual practice within the Community Strategy whereby they visit households according to the needs of the household.

Though we will not seek to establish HIV status of the participants, mothers in the intervention arms will be advised on how they should feed the child in case a mother is HIV positive, while the information materials given to both intervention and control arms will also stipulate information on feeding for HIV-exposed infants. Rigorous monitoring of the intervention will be done to ensure that the CHWs deliver the intervention as required. This monitoring will be done by an Intervention Monitor assisted by field assistants using monitoring tools developed for the project including mother’s diary and CHWs reporting tools. The CHWs will be expected to submit weekly reports of their activities to the intervention monitor. Additionally, close supervision of the activities of the CHWs will be done through regular spot checks and sit-in sessions by the intervention monitor. Further, a midline qualitative process evaluation will be done to assess if the intervention is happening as planned and assess areas that need improvement. The qualitative study will involve in-depth interviews and focus group discussion with mothers and CHWs.

### Assessment

#### *Outcome measures*

##### 

**Primary outcomes** The primary outcome measure is exclusive breastfeeding for six months.

This will involve determining the change in the rate of exclusive breastfeeding for the first six months. Mothers will be advised on what they need to do with regard to breastfeeding and how they should do it, including breast positioning and attachment and expressing and storing breast milk. This advice is expected to lead to self-efficacy with regard to breastfeeding, hence adherence to WHO guidelines on breastfeeding, resulting in improved rates of exclusive breastfeeding for six months. Data on breastfeeding practices will be collected longitudinally from birth every two months through an interviewer-administered questionnaire to the mother. Self-reported exclusive breastfeeding will be validated on a 10% sample using the stable isotope technique (dose-to-the mother of deuterium oxide) for breast milk analysis. The 10% sample will be randomly selected from the list of participating breastfeeding mothers. The procedure will be carried out once per mother-child pair, sampled before the child is aged six months.

##### 

**Secondary outcomes** Secondary outcomes include:

1. Breastfeeding and complementary feeding knowledge and attitudes according to WHO recommendations [9]. Data will be collected every two months following recruitment through an interviewer-administered questionnaire to the mother to determine change in knowledge, attitudes and practice with the intervention.

2. The duration of any breastfeeding in months.

This will include duration of any breastfeeding in months. Data will be collected longitudinally every two months following birth through an interviewer-administered questionnaire to the mother to determine if the child is still breastfeeding, and if so, whether they have started feeding on other foods/fluids other than breast milk; when they started on other foods/fluids; and if they stopped breastfeeding, when they stopped.

3. Complementary feeding practices.

This will focus on complementary feeding practices with regard to WHO recommendations, including timing of introduction of solid, semi-solid or soft foods; minimum dietary diversity; minimum meal frequency; minimum acceptable diet and consumption of iron-rich or iron-fortified foods [http://www.who.int/nutrition/publications/infantfeeding/9789241599290/en/] [9]. Data will be collected longitudinally every two months following birth through an interviewer-administered questionnaire to the mother to determine if the child has started feeding on foods/fluids other than breast milk and when they started, frequency of feeding, type of foods/fluids including dietary diversity through 24-hour recall, hygiene practices, and responsive feeding behaviours.

4. Nutritional status as determined by stunting, underweight, and wasting.

Interventions aimed at promoting optimal breastfeeding and complementary feeding practices have been found to reduce malnutrition among infants and young children [35]. It is therefore expected that the proposed intervention will have an effect on the levels of stunting, underweight and wasting. Anthropometric measurements: weight, length/height and mid-upper arm circumference will be collected on the child every two months during the follow-up period. All anthropometric measurements will be carried out according to standard procedures [36]. For determination of underweight, stunting, and wasting, weight-for-age z-scores (WAZ), height-for-age z-scores (HAZ) and weight-for-height z-scores (WHZ), respectively, will be generated using the World Health Organization (WHO) 2006 growth standards [37]. Stunting will be determined as HAZ < -2, underweight as WAZ < -2 and wasting as WHZ < -2 [38].

5. Morbidity from diarrhea.

Evidence indicates that breastfeeding is preventive against infections such as rotaviral diarrhoea [39]. It is therefore expected that promotion of exclusive breastfeeding would impact the rate of diarrhoea morbidity. Data on diarrhea morbidity in the last two weeks for the child will be collected longitudinally through an interviewer administered questionnaire to the mother every two months during the follow-up period.

6. Cost-effectiveness. Data on the cost of implementing the intervention from the provider perspective will be obtained from the project’s administrative records at the end of the study. By combining the cost and the effectiveness data, we will calculate the average cost-effectiveness ratio and the incremental cost effectiveness ratio.

### Other data to be collected

Other data to be collected will include:

1. Baseline data; collected at recruitment before the commencement of the intervention in both the control and intervention arms. Baseline data will include:

1.a. Knowledge, attitudes and practices regarding maternal and infant feeding.

1.b. Maternal anthropometric measurements.

1.c. Household food security.

1.d. Maternal socio-demographic characteristics and socio-economic status.

2. Child motor development (self-reported by the mother using questionnaire); updated every two months during the follow-up period after delivery.

3. Dietary intake for the mother collected every two months during the follow-up period through an interviewer administered questionnaire to the mother.

4. Maternal weight, monitored every two months during pregnancy and in the first year after delivery.

5. Contextual data, including morbidity from common childhood illnesses, antenatal care, place of delivery, vaccination, household food security, water and sanitation and hygiene practices will be collected through an interviewer administered questionnaire to the mother; updated every two months (where relevant) during the follow-up period.

### Before, during and after qualitative studies

In a formative study, in-depth interviews (n = 20), key informant interviews (n = 10) and focus group discussions (n = 10) will be conducted before the beginning of the intervention. In-depth interviews and focus group discussions will be conducted with women of reproductive age who are either pregnant, breastfeeding or have ever breastfed. Additional focus group discussions will be conducted with CHWs and village elders. Key informant interviews will be conducted among health care providers, traditional birth attendants and community leaders. The interviews will be conducted to establish the knowledge, attitudes and practices regarding breastfeeding and complementary feeding and to establish the local contexts and norms, which contribute towards decision-making for MIYCN practices. This information will inform the design of the intervention quantitative tools for assessing the main study outcomes.

As part of process evaluation, qualitative data will also be collected during the intervention and at the end of intervention to document experiences of adhering to optimal MIYCN practices emphasized in the intervention, and satisfaction with the intervention, challenges and enabling factors, and recommendations for change/future practice. This will be collected through in-depth interviews with mothers (approximately seven interviews), and focus group discussions with CHWs (two focus group discussions) at each point in time.

### Statistical analysis

Determination of change for the primary outcome (exclusive breastfeeding) will be done at month six. Difference in duration of exclusive breastfeeding will also be determined longitudinally up to six months between the intervention and control groups. Comparison for other outcomes such as morbidity from diarrhea and malnutrition will be done longitudinally at around month three, six, nine and twelve. Analyses will involve a comparison of differences in the intervention and control groups with regard to the primary outcome (exclusive breastfeeding) and secondary outcomes (other infant feeding practices, diarrhea morbidity and nutritional outcomes), controlling for baseline measures (for example, previous breastfeeding practices) and prognostic factors (for example, Socio-economic status (SES) and household food security) if randomization of the clusters does not control for differences in these factors at baseline. Statistical methods to determine change will include analysis of covariance (ANCOVA) for continuous variables (for example, duration of exclusive breastfeeding and any breastfeeding and multinomial logistic regression analysis for categorical variables (that is, whether exclusively breastfed for six months or not) [40]. Additionally, survival analysis (proportional hazard analysis) for variables measured longitudinally (for example, duration of breastfeeding and malnutrition and multilevel modelling for repeated measures such as diarrheal morbidity will be applied. Intention-to-treat analysis [41] will be applied as appropriate. Efforts will be made to maximize follow-up of participants, using the NUHDSS framework to minimize missing information. Quantitative data analysis will mostly be done using Stata version 12.1. (StataCorp LP, College Station, Texas, USA). (See Table 2 for detailed outline of data analysis plan).

**Table 2:** Data analysis outline

**Study objective**	**Outcome variables**	**Independent variable**	**Control variables**	**Type of analysis**
1. To determine the effect of the intervention on breastfeeding and complementary feeding knowledge, attitudes and practices among mothers of infants	1. Knowledge and attitude levels on breastfeeding and other infant feeding practices	Intervention status	SES^1^, food security, maternal demographic characteristics, antenatal care child morbidity	Multinomial logistic regression for rates and survival analysis for duration
	2. Breastfeeding practices, including initiation of breastfeeding within one hour of birth, exclusive breastfeeding (for first 6 months) and duration of exclusive and any breastfeeding			
	3. Complementary feeding practices for example, optimal timing of initiation of complementary feeding and minimum dietary diversity, minimum meal frequency, and minimum acceptable diet			
2. To determine the effect of the intervention on nutritional outcomes among infants;	1. Stunting	Intervention status	SES, food security, maternal demographic characteristics, child morbidity, antenatal care, vaccination status	Multinomial logistic regression
	2. Underweight			
	3. Wasting			
3. To determine the effect of the intervention on morbidity from diarrhoea among infants	Diarrhoea morbidity	Intervention status	SES, food security, maternal demographic characteristics, child morbidity, antenatal care, vaccination status	Multilevel modeling
4. To determine the experiences, facilitating and limiting factors associated with the intervention	1. Client satisfaction	Not applicable	Not applicable	Descriptive (Outcome 1) Qualitative-thematic analysis (outcomes 2 and 3)
	2. Experiences			
	3. Limiting and enabling factors			
5. To assess the cost-effectiveness of the intervention	Cost-effectiveness	Not applicable	Not applicable	Cost-Effective Analysis

^1^SES=Socio-economic status.

### Economic evaluation

An economic evaluation will be conducted to estimate the incremental cost-effectiveness of the personalised, home-based counseling compared to usual care, from the perspective of the provider. Cost-effectiveness will be determined with regards to cases of diarrhea morbidity and malnutrition averted using Cost Effective Analysis model (CEA) [42].

### Qualitative data analysis

Qualitative data will be transcribed verbatim and coded in NVIVO 10. Coding of the transcripts will be undertaken with themes being identified, for example, regarding breastfeeding and infant feeding beliefs and norms, with attention to contradiction and diversity of experiences and attitudes. Analysis will be conducted using a constant comparative method to identify themes and their repetitions and variations [43,44].

### Ethical considerations

Ethical approval has been granted by the Kenya Medical Research Institute (KEMRI), a recognized Ethical Review Committee, approved by the Government of Kenya. The investigators will uphold the fundamental principles regarding research on human subjects: respect for persons, beneficence and justice. For all data collection activities, informed consent will be sought from the eligible participants following full disclosure regarding the study before data collection is done. Proxy consent for children will be obtained from mothers.

## Discussion

This paper describes the protocol for a cluster-randomised trial whose aim is to determine the effectiveness of personalised home-based counselling of urban poor women by community health workers on breastfeeding and other infant feeding practices. Breastfeeding and optimal infant feeding promotion is an important intervention for child-survival; however, it is not yet clear which strategies are the most effective. Studies have indicated the effectiveness of counselling programs within primary health care in improving breastfeeding and other infant feeding practices particularly in high- and middle-income countries, but little evidence of this exists for low-income countries [30,45-48].

The BFHI, a WHO and UNICEF global program launched in 1991 to protect, promote, and support breastfeeding in maternity wards has been found effective in improving breastfeeding practices particularly in high- and middle-income countries [49-52]. Of concern, however, is the fact that the BFHI mainly focuses on promoting breastfeeding in the hospital setting around the time of delivery, yet in some countries, very few women deliver in health facilities [18]. Studies have found that while interventions that involve counselling/support at the health facility level are effective, there is evidence to suggest that combining hospital-based counselling/support with home-based visits is more effective. For example, in a study done in Brazil that compared two systems of delivery of breastfeeding counselling/support (a purely hospital-based system and a hospital based system coupled with home-visits), the hospital-based intervention achieved a high rate (70%) of exclusive breastfeeding during the hospital stay, but this was not sustained after discharge from hospital; at 10 days, this rate had dropped to 30%. The rate of exclusive breastfeeding from 10 days to six months was higher in the group that received home visits (45%) compared to the group with only hospital-based intervention (13%) [53]. This study will provide further input into the effectiveness of home-based counselling in an area with very limited health care access.

The timing of the counselling/support has been found to be important. Prenatal and postnatal breastfeeding counselling interventions, whether alone or in combination, have been found effective in improving breastfeeding practices including duration of exclusive breastfeeding and any breastfeeding [47,48]. In a systematic review of effectiveness of breastfeeding interventions that involved 38 trials, prenatal breastfeeding interventions significantly increased the rate of any short-term breastfeeding (1 to 3 months) rate by 39% compared to the usual care while combined prenatal and postnatal interventions significantly increased both the rates of intermediate (4 to 5 months) and long-term (6 to 8 months) any breastfeeding compared to usual care by 15% and 33%, respectively. Postnatal interventions significantly increased the rates of short-term exclusive breastfeeding (1 to 3 months) by 21%. In another systematic review that involved 20 trials, only interventions with a post-natal component were found to be effective in improving breastfeeding practices whereas there was no evidence to suggest effectiveness of antenatal counselling/support [48]. This study, which involves home-based personalised counselling/support during pregnancy and one year following delivery, will provide further evidence on the effectiveness of combined antenatal and postnatal counselling/support.

The mode of delivery of the intervention has also been found to be important. Some studies have indicated that interventions involving face-to-face contact are more effective than those not involving a face-to-face, contact such as through telephone [48]. Additionally, while interventions involving a component of lay counselling/support or structured education or professional counselling/support alone or in combination are effective in improving breastfeeding practices [30,47,54], some evidence further suggests that interventions that include a component of lay support/counselling are more effective than interventions involving structured education or professional support/counselling only [47]. Other evidence indicates that interventions involving professional support are effective in improving any breastfeeding but not exclusive breastfeeding, while lay support is effective in improving exclusive breastfeeding but has no effect on any breastfeeding [48]. This indicates that the effect of these interventions is not straightforward and more research is needed. The effectiveness of CHWs in health care delivery, particularly in child-survival programs has been indicated [46,55,56]. A study involving a non-randomised design in rural Kenya to determine the effectiveness of the Government’s Community Strategy (that involves use of CHWs to promote health in the community) found that the strategy improved the rate of exclusive breastfeeding from 20% to 52% [46], although the robustness of the methods used is questionable because it was a pre-test and post-test non-randomised, interventional study (with no control). This study will provide more insights into the effectiveness of personalised, face-face counselling/support using CHWs in promoting breastfeeding and other infant feeding practices in a setting with very limited access to professional counselling in urban slums in Kenya.

Other than just assessing the effectiveness of the interventions on breastfeeding and other infant feeding practices, this study will also determine the effectiveness of the intervention on other outcomes including morbidity from diarrhea and nutritional outcomes [48,49,52]. Studies elsewhere have indicated the effectiveness of interventions aimed at promoting breastfeeding on these outcomes. A good example of a study where these effects have been studied is the PROBIT (Promotion of Breastfeeding Intervention Trial) study in Belarus in 1996 to 1997 that involved slightly over 17,000 mother-child pairs. That study showed a significant reduction in the risk of gastrointestinal infections and a positive effect on growth of infants. The prevalence of gastro-intestinal infections was 9% in the intervention group compared to 13% in the control group (OR = 0.6; CI: 0.40-0.91; *P* <0.05). Likewise, infants in the intervention group were 106 g and 89 g heavier; and 0.50 and 0.46 cm taller at three and six months respectively compared to children in the control group (*P* <0.05) [49,52]. The effects may be related to optimal breastfeeding and other infant feeding practices on growth and morbidity.

Some limitations to this study may include bias in reporting of the primary outcome (exclusive breastfeeding). To counter this, objective breast milk analysis using the stable isotope technique will be used. It is possible that even with randomization of clusters, there will be difference in the baseline measures in the intervention and control group by chance. Analysis methods to be adopted will control for baseline differences. Additionally, given high mobility in the urban slums, some of the participants may be lost to follow-up, while some may also move to alternative group clusters. To counter this, intention to treat analysis will be adopted, while an allowance for loss to follow-up has been included in the sample size determination.

## Conclusions

The importance of breastfeeding and optimal infant feeding promotion in child-survival cannot be overemphasized. Identifying effective strategies for promotion of optimal infant feeding is of utmost importance. The results from this trial will provide evidence regarding the effectiveness of personalised, home-based counselling of mothers during pregnancy and within the first year after delivery on breastfeeding and other infant feeding practices, morbidity from diarrhea, and nutritional status among infants, and the cost-effectiveness of the intervention. This is expected to inform policy and practice regarding child survival particularly for the urban poor populations. It is also expected to inform the roll-out of the BFCI in Kenya and other developing countries where it is under consideration, which goes beyond the BFHI to promote optimal breastfeeding and other infant feeding practices at the community level.

## Trial status

The trial is currently recruiting pregnant women on a rolling basis and collecting baseline and follow-up data. Recruitment is expected to end in December 2013.

## Abbreviations

ANCOVA: Analysis of covariance; APHRC: African Population and Health Research Center; BFCI: Baby-Friendly Community Initiative; BFHI: Baby-Friendly Hospital Initiative; CEA: Cost effective analysis; CHWs: Community health workers; CUs: Community units; DALYs: Disability-adjusted life years; KEMRI: Kenya Medical Research Institute; ICC: Intra-cluster correlation; IYCN: Infant and young child nutrition; LIMCs: Low- and middle-income countries; MDGs: Millennium Development Goals; MIYCN: Maternal, infant and young child nutrition; NUHDSS: Nairobi Urban Health and Demographic System; SES: Socio-economic status; SSA: Sub-Saharan Africa; TBA: Traditional birth attendant; UNICEF: United Nations Children’s Fund; WHO: World Health Organization.

## Competing interests

The authors declare that they have no competing interests.

## Authors’ contributions

EWK-M designed the study, wrote the manuscript and approved the manuscript for submission. CK, ACE, SAN, PG, and NJM designed the study, gave guidance on writing the manuscript, reviewed the manuscript and approved the manuscript for submission. FW, MW, PM, and RNM designed the study, reviewed the manuscript and approved the manuscript for submission. All authors read and approved the final manuscript.

## Supplementary Material

Additional file 1 Sample size determination. Click here for file
