# Supplementary material for: Effectiveness of personalised, home-based nutritional counselling on infant feeding practices, morbidity and nutritional outcomes among infants in Nairobi slums: study protocol for a cluster randomised controlled trial
Source: Trials. 2013 Dec 27;14:445. doi: 10.1186/1745-6215-14-445 (PMC3879433; doi:10.1186/1745-6215-14-445)
Supplement: Additional file 1 — Sample size determination. [file 1745-6215-14-445-S1.docx]

**Additional file 1: Sample Size Determination**

The sample size determination was done considering the study design of cluster randomisation since villages in the study sites will be randomised to either control or intervention [[1](#_ENREF_1)]. The total sample size required is estimated by first estimating unadjusted sample size using traditional formula for comparing two proportions. This is given as;


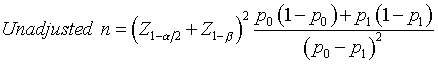


Where Zα/2 = the critical value from the Standard Normal Distribution corresponding to α significance level (i.e. Z0.975 = 1.96 for a 5% level of significance); Zβ = the critical value from the Standard Normal Distribution giving β = 20% of the upper tail i.e. 80% power (Z0.8 = 0.84);

P0 = proportion with attribute of interest in control group; P1= proportion with attribute of interest in intervention group;

Assuming prevalence of exclusive breastfeeding of 2% in the control group [[2](#_ENREF_2)] and expected prevalence of 12% in the intervention group, unadjusted sample size of 196 mother-child pairs in both groups is required. Secondly, the sample size obtained using traditional formula was adjusted to account for intra-cluster correlation as;


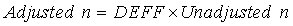


Where DEFF is the design effect which is an inflation factor to account for clustering.

DEFF= 1+ (m-1)*ICC, where m= average size of cluster & ICC=intra-cluster correlation coefficient.

An ICC of 0.05 for breastfeeding behaviour among mothers of infants was used based on previous research in the study setting [[3](#_ENREF_3)]; and an m of 45 (based on expected number of participants per village using the unadjusted n); hence DEFF=3.2

Therefore, a minimum adjusted sample size of 622 was estimated for both arms. We allowed for 20% potential attrition based on experience, to get a sample size of 777 (approx. 780) for both arms of the study.

References

1. Chan YH: **Randomised controlled trials (RCTs)--sample size: the magic number?** *Singapore Med J* 2003, **44**(4):172-174.

2. Kimani-Murage E, Madise N, Fotso J-C, Kyobutungi C, Mutua, K, , Gitau T, Yatich N: **Patterns and determinants of breastfeeding and complementary feeding practices in urban informal settlements, Nairobi Kenya**. *BMC Public Health* 2011, **11**(396).

3. Fotso JC, Madise N, Baschieri A, Cleland J, Zulu E, Mutua MK, Essendi H: **Child growth in urban deprived settings: does household poverty status matter? At which stage of child development?** *Health Place* 2012, **18**(2):375-384.
